# Plum (*Prunus domestica*) Trees Transformed with Poplar *FT1* Result in Altered Architecture, Dormancy Requirement, and Continuous Flowering

**DOI:** 10.1371/journal.pone.0040715

**Published:** 2012-07-30

**Authors:** Chinnathambi Srinivasan, Chris Dardick, Ann Callahan, Ralph Scorza

**Affiliations:** United States Department of Agriculture, Agricultural Research Service, Appalachian Fruit Research Station, Kearneysville, West Virginia, United States of America; Kansas State University, United States of America

## Abstract

The *Flowering Locus T1* (*FT1*) gene from *Populus trichocarpa* under the control of the 35S promoter was transformed into European plum (*Prunus domestica* L). Transgenic plants expressing higher levels of FT flowered and produced fruits in the greenhouse within 1 to 10 months. FT plums did not enter dormancy after cold or short day treatments yet field planted FT plums remained winter hardy down to at least −10°C. The plants also displayed pleiotropic phenotypes atypical for plum including shrub-type growth habit and panicle flower architecture. The flowering and fruiting phenotype was found to be continuous in the greenhouse but limited to spring and fall in the field. The pattern of flowering in the field correlated with lower daily temperatures. This apparent temperature effect was subsequently confirmed in growth chamber studies. The pleitropic phenotypes associated with *FT1* expression in plum suggests a fundamental role of this gene in plant growth and development. This study demonstrates the potential for a single transgene event to markedly affect the vegetative and reproductive growth and development of an economically important temperate woody perennial crop. We suggest that *FT1* may be a useful tool to modify temperate plants to changing climates and/or to adapt these crops to new growing areas.

## Introduction

The flowering and dormancy requirements of temperate trees limit the climactic ranges under which they can be grown and renders them susceptible to climate change. Genetic engineering offers new possibilities for developing more resilient and productive tree-based agricultural systems by manipulating these relatively fixed characters. Engineering trees with altered dormancy, flowering, and architectural characteristics should enable substantial advancements in breeding time, productivity, and sustainability. Over the past decade, the molecular mechanisms of flower formation in plants have been deciphered to a great extent. In the model plant *Arabidopsis* a number of genes have been identified that are directly or indirectly associated with flower induction and both their interactions and modes of action have been established (recently reviewed by Srikanth and Schmid, 2011) [1]. Up or down regulation of these genes have potential practical applications as they have been shown to promote or repress flowering in a number of plant species [2] including trees [3]–[7].

In trees, the time to maturity is critical to the breeding cycle where the juvenility period prior to the first flowering can range from three to over 20 years for some species. Successful early flowering in trees has been obtained utilizing *Flowering Locus T* (*FT*) genes or orthologues of a MADS-BOX gene called *FRUITFULL* (*FUL*). Utilizing either *FT1* isolated from *Populus trichocarpa)* or *FT2* from *Populus deltoids-* male poplar hybrid *P. trem*ula x P. *tremuloides* flowered as early as four weeks post-transformation when over expressing *PtFT1*, or after one year when expressing *FT2* [4] [8]. Recent results suggest that FT2 is also involved in vegetative growth in poplar [9] while in apple FT2 controls both fruit development and vegetative growth [10]. Both FT1 and FT2 coordinate the repeated cycles of vegetative and reproductive growth in woody perennial trees [9]–[10]. The apple cultivar ‘Pinova’ was induced to flower early by over expressing a silver birch (*Betula pendula Roth*) floral meristem identity MADS-box gene (*BpMADS4*) which is similar to *FUL* [6]. These transgenic apple plants flowered within 13 weeks after transformation. Pollination of the flowers with *Malus fusca* pollen produced small but otherwise normal fruits and seeds. Breeding applications using these trees are currently underway [11]–[12]. The apple *FT* gene was also able to induce early flowering in tissue culture in apples and in poplar [5]. In citrus, constitutive expression of citrus *FT* (*CiFT*) in trifoliate orange (*Poncirus trifoliata*) induced flowering as early as three months following transfer to the greenhouse from tissue culture and produced morphologically normal fruit with viable seeds [3]. A table of fruit tree species in which precocious flowering has been induced is shown (Table 1).

**Table 1:** Ectopic expression of flower inducing genes in woody perennial fruit trees.

Gene Construct	Source	Expressed In	Early Flowering	Reference
35S::LFY	*A. thaliana*	*Citrus sinensis x Poncirus trifoliata*	Yes	Pena et al 2001
		*Malus x domestica Cv* ‘Pinova’	No	Flachowsky et al 2010
35S::AP1	*A. thaliana*	*Citrus sinensis x Poncirus trifoliate*	Yes	Pena et al 2001
		*Malus x domestica Cv ‘*M26’	No	Zhu et al 2009
		*Fortunela crassifolia Cv* ‘Meiwa’	Yes	Duan et al. 2010
35S::BpMADS4	*Betula pendula*	*Malus x domestica Cv ‘Pinova’*	Yes	Flachowsky 2007
35S::CiFT	*Citrus unshiu*	*Pyrus communis Cv* ‘La France’ & ‘Balade’	Yes	Matsuda et al 2009
35S::MdFT	*Malus domestica*	*Malus domestica Cv* ‘Pinova’	Yes	Trankner et al 2010

Surprisingly, some flowering regulators have also been shown to influence dormancy in trees suggesting a close relationship between these two biological functions. In temperate trees, dormancy is induced by shortened day length, cold temperatures, or both. Once dormant, buds (both floral and vegetative) will not complete development until they experience a certain period of cold temperatures, commonly expressed as a number of chilling hours. Poplar trees over-expressing *PtFT1* were insensitive to changes in day length and did not enter short-day induced dormancy [4]. Likewise, RNAi down-regulation of the poplar flowering regulator *TERMINAL FLOWER1* (*TFL1*) homologues *CENTRORADIALIS1* (*CEN1*) or *CEN2* reduced the time to dormancy release [13]. Over-expression of the birch *FRUITFULL-like* MADS-box gene *BpMADS4* blocked senescence and altered dormancy in poplar [14]. Collectively these studies highlight the opportunities and challenges to simultaneously manipulate flowering and alter climate tolerance in tree species. Practical application of these technologies is now just beginning to emerge.

Temperate fruit crops and other deciduous tree species require specific temperature regimes for proper vegetative growth and reproductive development. Breeding for reduced chilling requirement or enhanced cold tolerance has expanded production ranges for some temperate crops; however, this is limited by the relatively narrow genetic variability for these traits. In an extreme example, a peach variety, ‘Evergrowing’ (Evg), was identified which did not have a chilling requirement [15]. The *Evg* locus was mapped to a cluster of MADS-BOX genes the functions of which have not yet been elucidated [16]. Peach trees carrying the *Evg* gene can be grown in the tropical regions of Mexico though they still require a period of rest which in practice is established by defoliation [17]–[18]. Here we report the development and characterization of early flowering *Prunus domestica* lines engineered to over-express the poplar *FT1* gene. These lines show potential for accelerating breeding cycles, reducing juvenility time, and adaptation to climate regimes not normally associated with temperate fruit species.

## Results and Discussion

**Figure 1:**
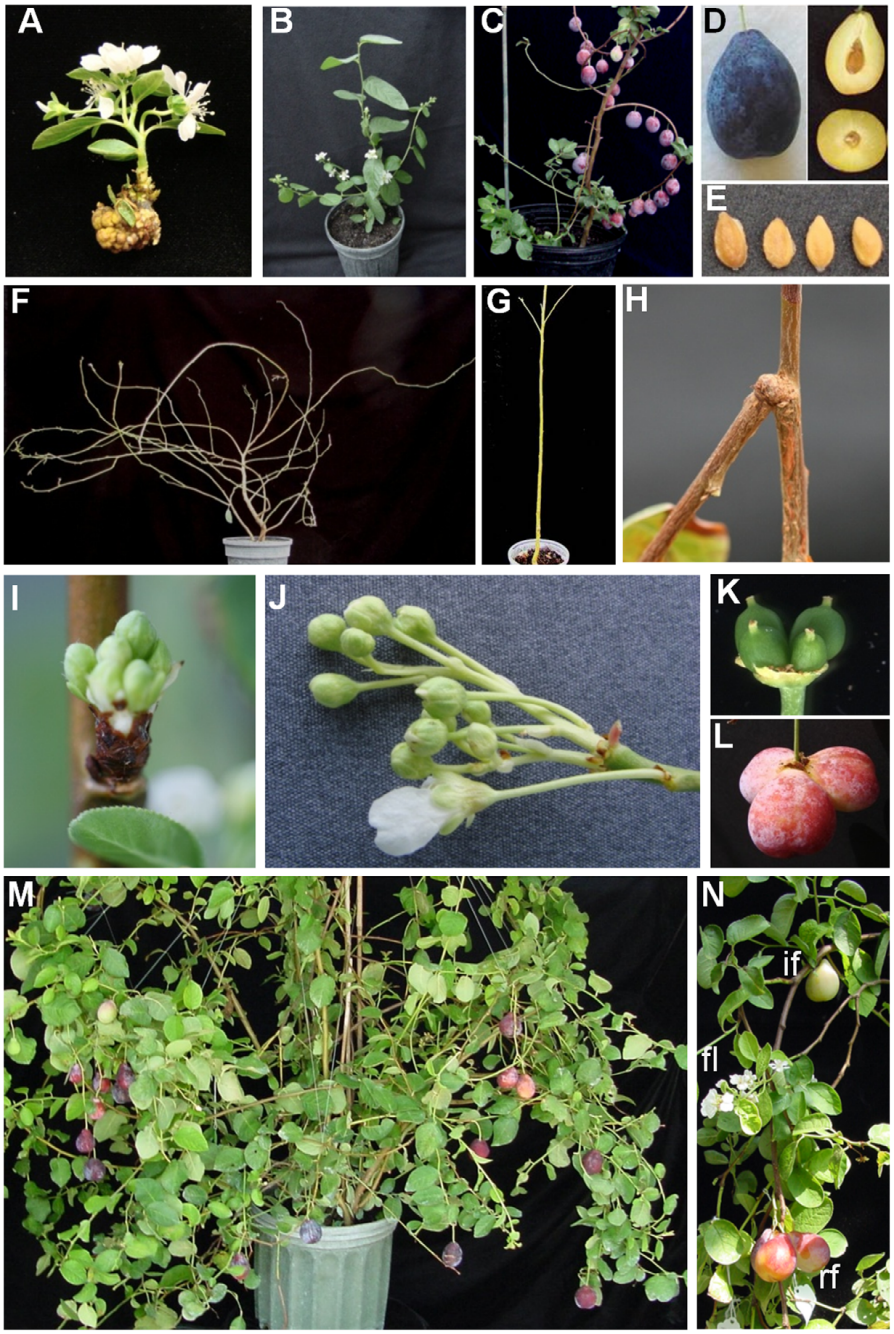
Phenotypic characteristics of FT plums. A regenerating FT plum line *in vitro* (A). Flowers on FT plum line #3 two months after planting in soil (B). One year old FT plum line #157 with ripe fruits (C). Cross and longitudinal sections of FT plum fruit (D) and dried seeds (stones) (E). Shrub architecture of one year old FT plum line #157 canopy (F) normal plum (G). Example of a downward branch angle in FT plum line #157 (H). Multiple flowers emerging from a single bud (I) and a resulting flower panicle (J). A flower is shown in which sepals, petals and anthers were removed revealing four carpels (K) that when pollinated produce multiple fruits (L). FT plum line #3 with ripe fruit after 28 months of continuous growth in the greenhouse (M). Flowers (fl), immature fruit (if), and ripening fruits (rf) on a single FT plum plant (N).

European plum (*Prunus domestica* L) is a temperate tree crop that has a juvenility phase of three to seven years. We engineered ‘BlueByrd’ plum trees with the *FT1* gene from *Populus trichocarpa* under the control of the 35S promoter [4] [12]. Eight plum plantlets were observed to flower *in vitro* (Fig. 1A) but were unable to be rooted as they lack vegetative meristems. A total of 196 lines derived from 56 plum hypocotyl slices were established in the greenhouse. All were confirmed by PCR to be carrying the *PtFT* gene (data not shown). Flowering occurred in 32 out of 196 lines within 1 to 10 months after transfer to soil (Fig. 1B). Sixteen additional lines flowered within the next several months. Hand pollination of flowers led to significant fruit set. Fruit development appeared normal as they ripened within 5 months, a similar time frame to the parent in the orchard. Ninety ripe fruits were analyzed for phenotypic properties. The fruits ranged in size between 28 to 37 mm in diameter, 13.4 to 33.9 g in weight and had 10° to 13° Brix. These values were all lower than the parental line ‘Bluebyrd’ (Fig. 1C). The colors of the fruits ranged from various shades of red to the deep purple of the parental line. Stones and seeds also developed normally but they were smaller than the orchard grown plum seeds (Fig. 1D, 1E). The observed variation in phenotypic parameters was expected for a seedling population and not directly attributable to FT expression.

To determine whether the early flowering trait could be transferred to the next generation, seeds from fruits set by three FT plum lines (PtFT3, PtFT34, and PtFT157) were germinated. Germinated seedlings were scored for flowering for 18 months following germination. The seedlings derived from lines PtFT3 and PtFT34 segregated for the early flowering phenotype in a 1∶1 ratio (6/16 and 17/33, respectively) and had the characteristic flowering phenotypes of the parental transgenic lines. Line PtFT34 showed a lower than expected ratio of flowering phenotypes (8/32). PCR confirmed that eight additional PtFT34 seedlings contained the FT gene but had not flowered at the time of scoring. This result confirmed the 1∶1 genetic segregation as the lack of observable phenotype was likely a consequence of the weaker flowering exhibited by line PtFT34. In total, the data established that these lines can be used for rapid plum breeding.

In addition to the premature flowering phenotype, the FT plum lines expressed pleiotropic phenotypes that rendered them different from normal plum trees. They did not grow with a typical upright tree growth habit, but instead became highly branched resulting in a shrub-type growth habit (Fig. 1F, 1G). This shrub like growth habit was the result of: 1) loss of apical dominance that was sometimes due to the production of a terminal inflorescence, 2) trunk and lateral branches expressing a curved growth pattern, 3) lateral branches with occasionally weaker attachment to the main trunk that could separate and re-heal resulting in downward angled branches (Fig. 1H), and 4) the growth of long lateral branches from vegetative buds at the trunk. Effects on tree growth architecture, especially increased lateral branching, were also reported in citrus trees over-expressing *FT* and poplar trees with down-regulated *CEN/TFL1* [3] [13].

Flowering architecture was also altered in FT plums. Plum flower buds normally develop in early summer on one year old stems flanking a vegetative bud at each node. The following spring single flowers or umbels of two to six flowers emerge from each floral bud. Unlike control plums, FT plums continually formed flower buds, and the timing of bud break or bud fate was not predictable. Almost any combination of flowers and vegetative shoots per node were present on any given plant. Instead of single flowers or umbels, some buds gave rise to atypical flower panicles which are not known to occur in *Prunus* species (Fig. 1I and 1J). Individual flowers sometimes produced multiple carpels which when pollinated gave rise to 2–5 fruits per flower each containing a viable seed (Fig. 1K and 1L).

To determine whether the expression level of *PtFT1* was correlated with the early flowering and architecture phenotypes observed in individual *PtFT1* lines, the relative expression of *PtFT1* was measured from leaf samples taken from both flowering and non-flowering transgenic lines. The selected lines included those that also showed variations in architecture (Fig. 1, Table 2). Flowering lines consistently showed the highest levels of *PtFT1* gene transcripts (Table 2). In contrast, the architectural phenotypes of increased lateral branching (ie. bushy) were not correlated with higher *PtFT1* expression. Given that variations in the architecture ‘Bluebyrd’ plum seedlings are rare, it appears these traits require only low levels of *PtFT1* expression in contrast to flowering which was associated with higher *PtFT1* expression.

**Table 2:** Gene expression and copy ratio of PtFT1 in transgenic plums.

			*PtFT1*	*PtFT1*/4040^3^	
Line	Phenotype	Flowering	RNA^2^	Ratio	#Copies^4^
7	Normal	No	6%	0.1981	1
104	Normal	No	6%	0.7921	4
143	Bushy	No	7%	0.4185	2
110	Bushy	No	8%	0.9236	4
194	Bushy	No	10%	7.0710	multiple
114	Bushy	No	13%	0.2115	1
Control B	Normal	No	24%	0.0016	none
4	Normal	No	24%	0.1730	1
107	Bushy	Yes	46%	0.9324	4
183	Bushy	Yes	53%	0.4677	2
157	Bushy	Yes^1^	68%	0.9583	4
3	Bushy	Yes^1^	81%	1.6942	multiple
103	Bushy	Yes^1^	94%	0.5999	3
117	Bushy	Yes	96%	2.0257	multiple
126	Bushy	Yes	100%	0.5778	3
174	Bushy	Yes^1^	187%	0.9280	4
175	Bushy	Yes	271%	0.8153	4
188	Bushy	Yes	286%	3.1313	multiple

1 profusely flowering.

2 average relative amount of PtFT RNA relative to early flowering line 126.

3 ratio of real-time value of PtFT to single copy gene 4040.

4 copy number of PtFT insertion based on lowest significant ratio.

To determine if the copy number of inserted *PtFT1* genes correlated with the expression levels or the phenotypes, real-time PCR was used to determine the *PtFT1* transgene copy number. Copy number ranged from one to >4 copies (Table 2). When compared to the RNA transcript level, there was no correlation of copy number and RNA expression except that all the high expressing lines had at least two copies. Conversely, those that had only a single copy of PtFT1 did not flower (Table 2). Lack of strong correlation between gene copy number and flowering has also been observed in early flowering transgenic pears [7].

Unlike control plums which require dormancy, FT plums continually grew in pots, flowered, and produced fruits even after three years of continual cultivation in a temperature controlled greenhouse (18–24°C) (Fig. 1M). Flowers and fruits at all developmental stages could be found at all times on a single plant much like indeterminate tomato plants (Fig. 1N). These and the other unique characteristics of FT plums prompted us to closely examine their dormancy and flowering characteristics.

**Figure 2:**
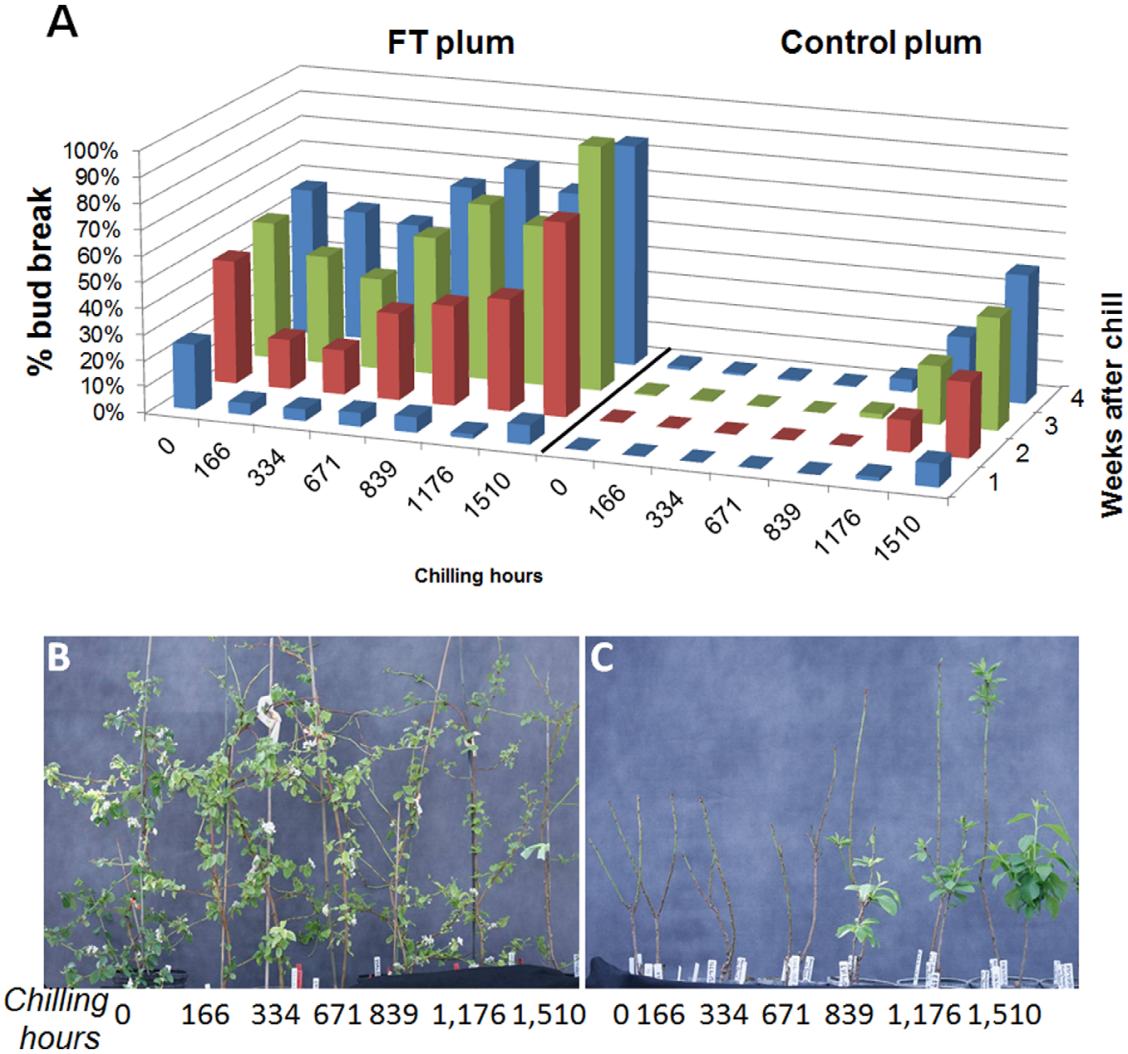
FT plums do not respond to cold treatment. Graph showing mean percentage of bud break (Y axis) after transfer to greenhouse upon selected intervals of chilling time (X-axis) measured for up to four weeks (Z-axis) (A).Images of cold treated FT line #34 (B) and normal (C) plums after indicated chilling times at 4°C.

A hallmark of temperate tree crops is their dependence on a period of dormancy for growth and reproduction. Cool weather and short day lengths in fall promote the onset of endodormancy which is associated with terminal bud formation, growth cessation, and freezing tolerance. Once endodormancy is established, a sufficient number of chilling hours (generally defined as hours exposed to 2–12°C) is required before efficient vernalization can occur [19]. For *P. domestica,* 1,000–2,000 chilling hours are typically needed to resume growth. We tested whether FT plums were responsive to low temperature by placing clonally propagated plants in cold storage at 5°C and then moving them to the greenhouse (18–24°C) at one week intervals. Once out of the cold, the rate of bud break was measured for four weeks. Results showed that FT plums had no chilling requirement as bud break rates did not significantly change between 0 and 1,500 hours of chilling unlike control plums which showed little or no bud break until they acquired over 1,100 hours of chilling (Fig. 2A-2C). Regardless of treatment, FT plums also showed a high rate of bud break relative to controls reaching 100% in some plants. These data indicate that FT plums do not enter a state of endodormancy upon cold treatment or alternatively have no chilling requirement after dormancy is established.

**Figure 3:**
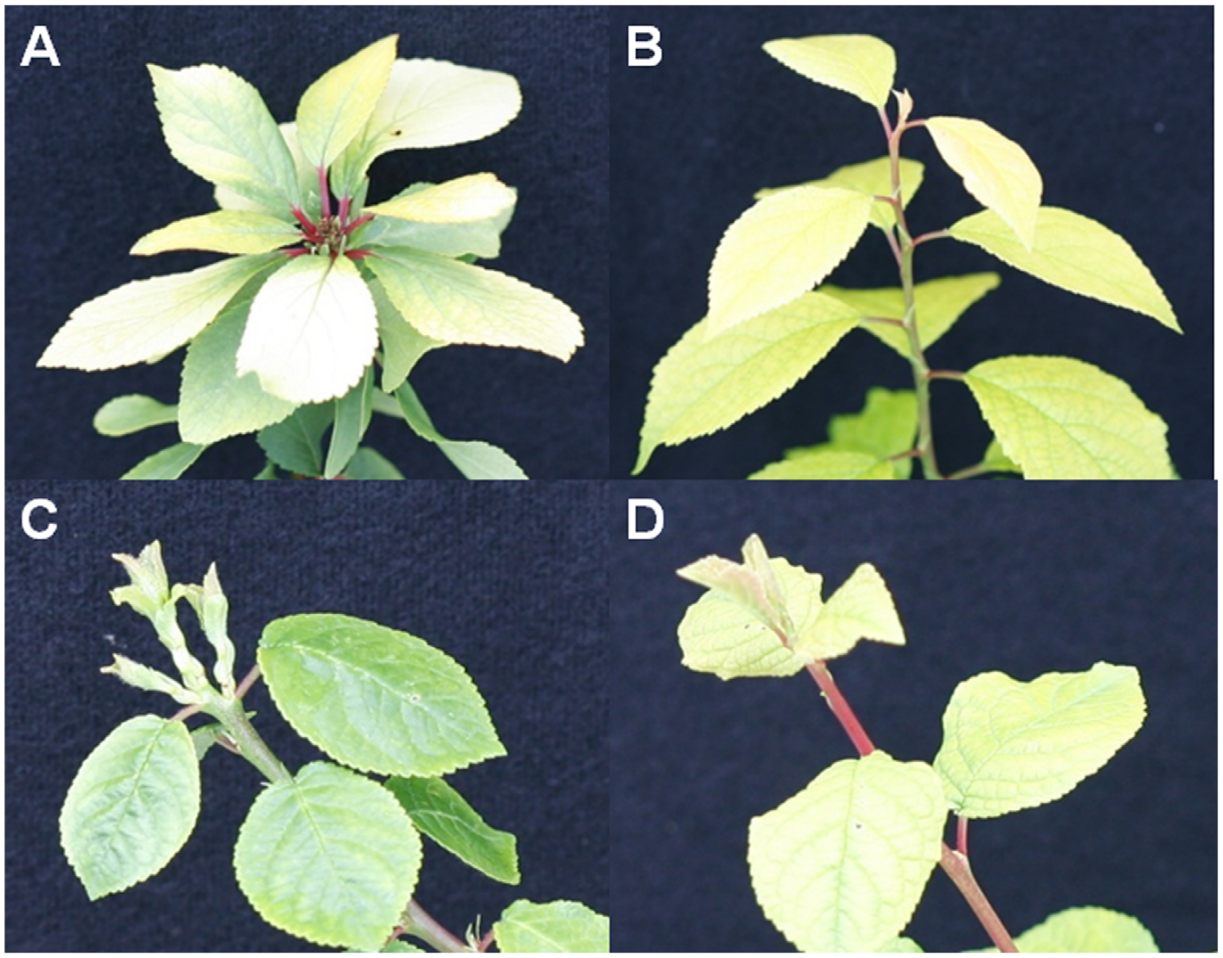
FT plums do not set terminal buds under short day lengths. Vegetative meristems from control plums showing terminal buds when grown under an 8 hr day length (A) but not under 16 hrs (B). Growth pause in FT plum line #34 grown under an 8 hr day length (C) and continued growth under 16 hr (D) days.

FT over-expressing poplar trees were previously shown to be insensitive to short-day induced dormancy [4]. To determine if FT plums showed a similar phenotype, six PtFT34 plants and six non-transgenic controls were grown under short day lengths (SD) (8 hr) and long (16 hr) day lengths (LD) at 21°C. After seven weeks under each condition, plants were exchanged (plants under LD were placed under SD and vice versa) to simulate a sudden change in day length, and terminal bud set was monitored after 14 days. The results demonstrated that FT plums did not set terminal buds as the control plants did (Fig. 3). The FT plants continued to flower and set new flower buds under both conditions. After the exchange, 0 out of 29 FT plum shoots shifted from LD to SD set terminal buds though some showed a brief pause in growth but did not cease growing as newly developed buds proceeded to break (Fig. 3). In contrast, 6 out of 18 control plum shoots ceased growth and developed terminal buds when shifted to SD conditions. Thus, FT plums appear to sense sudden changes in day length but do not enter a prolonged state of endodormancy.

Given their apparent inability to enter dormancy, FT plums were tested for survival in a natural winter environment. Four plants each from two different transgenic FT lines (#3 and #34) along with controls were planted in field plots in early summer of 2009. In late January, 2010, bud sticks from one year old branches were removed and assayed for survival. Bud survival rates were estimated to be 96% for control non-transgenic plums, 100% for PtFT34, and 84% for PtFT3. Local weather data indicated they experienced 2,631 chilling hours and had been exposed to temperatures between −5°C and −13°C for periods up to 48 hours suggesting that even though FT plums are incapable of entering into endodormancy they remain at least somewhat winter hardy in a U.S. mid-Atlantic climate. Forty five additional PtFT plum lines were planted in the field in the spring of 2011. All survived the 2011–2012 winter which again had temperatures as low as −13°C for periods up to 48 hours.

**Figure 4:**
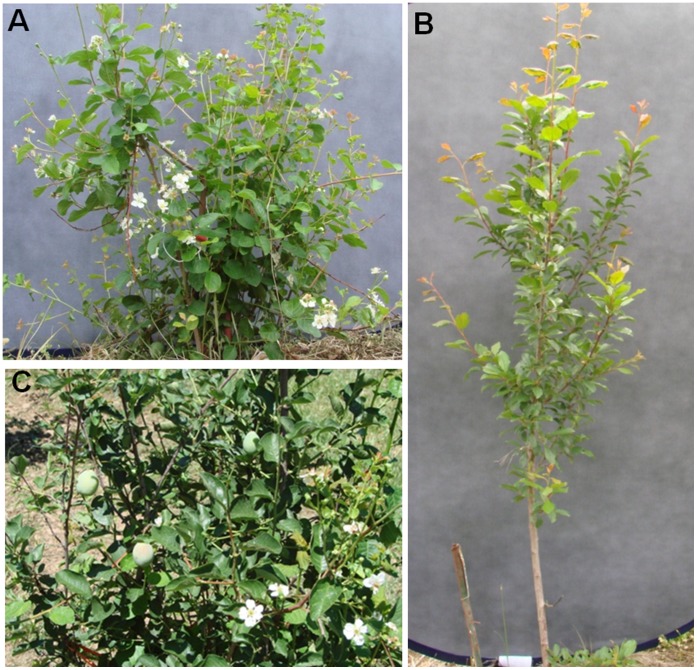
Growth habit, flowering, and fruiting of field grown FT plums. FT plum line #34 (120 cm in height) shown flowering six weeks after the normal anthesis period (first week in April) (A). A non-transgenic control plum (190 cm in height) of the same age that does not flower (B). Resumed flowering of FT plum line #34 in fall (last week of October) (C).

**Figure 5:**
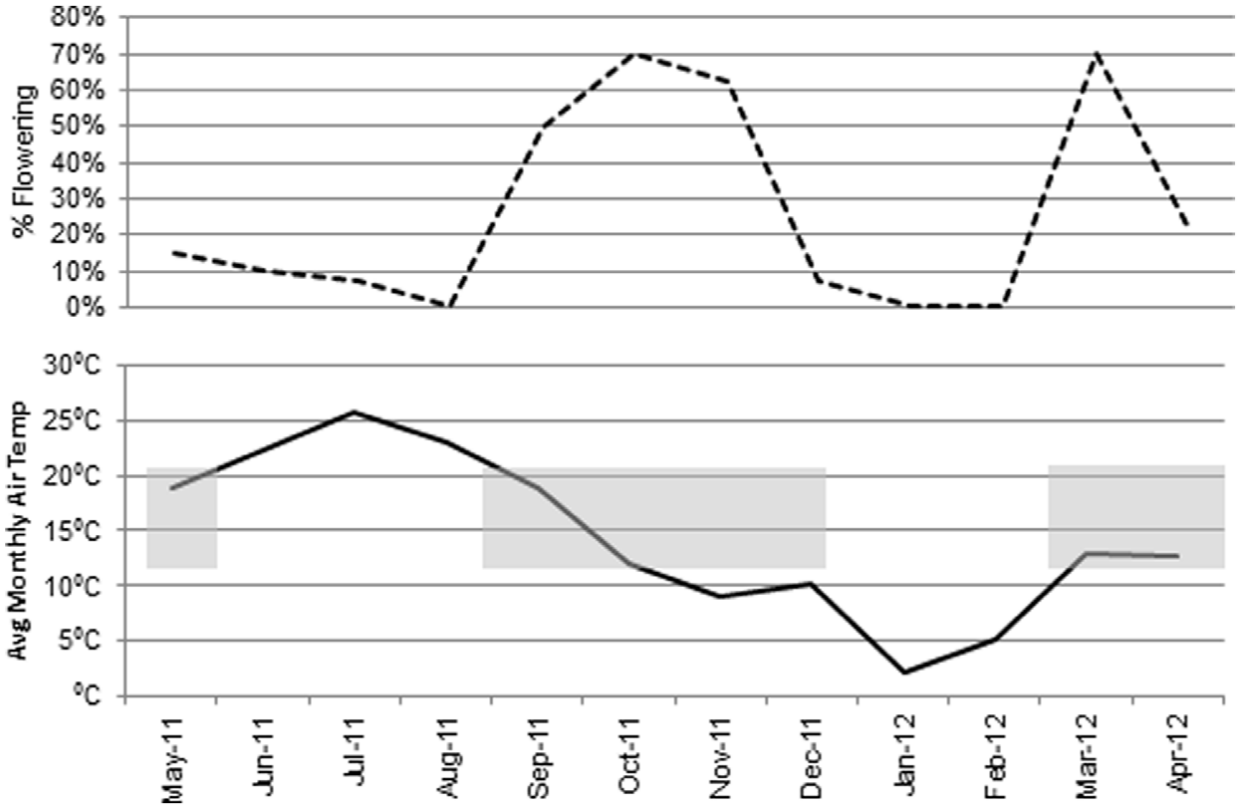
Flowering in field grown FT plums correlates with temperature. Graph showing the percentage of PtFT plums that flowered each month (top) and the average monthly air temperature (bottom). Monthly dates are shown on X-axis. Shaded gray bars indicate periods of significant flowering. Small black bar on X-axis indicates the observed flowering period of control plums.

To extend the bud survival data, field grown FT plum plants were evaluated weekly throughout 2011 for growth architecture and flowering patterns. PtFT3and PtFT34 showed significant growth architecture differences relative to controls (Fig. 4). FT plums displayed normal vegetative bud break and flowered in the Spring along with control plum trees. Unlike control plums which only produce flowers from overwintered buds, FT plums put out flowers both from overwintered flower buds and from buds developed on new growth. Similar to flowering under greenhouse conditions, panicle type-flowering structures were widely prevalent in the field. Control plums ceased flowering by mid-April but FT plums continued to flower for an additional 10–12 weeks into early summer. FT plums ceased flowering in July and August. They then initiated a second flush of flowering in late September. Fall flowering continued into the first week of December. Based on our previous finding that day length did not influence flowering, we examined the potential role of temperature. Average monthly temperatures were plotted against the observed flowering periods (Fig. 5). FT plums flowered when average temperatures were consistently above 8°C but below 22°C. A similar flowering pattern had also been observed in 2010 (data not shown).

**Figure 6:**
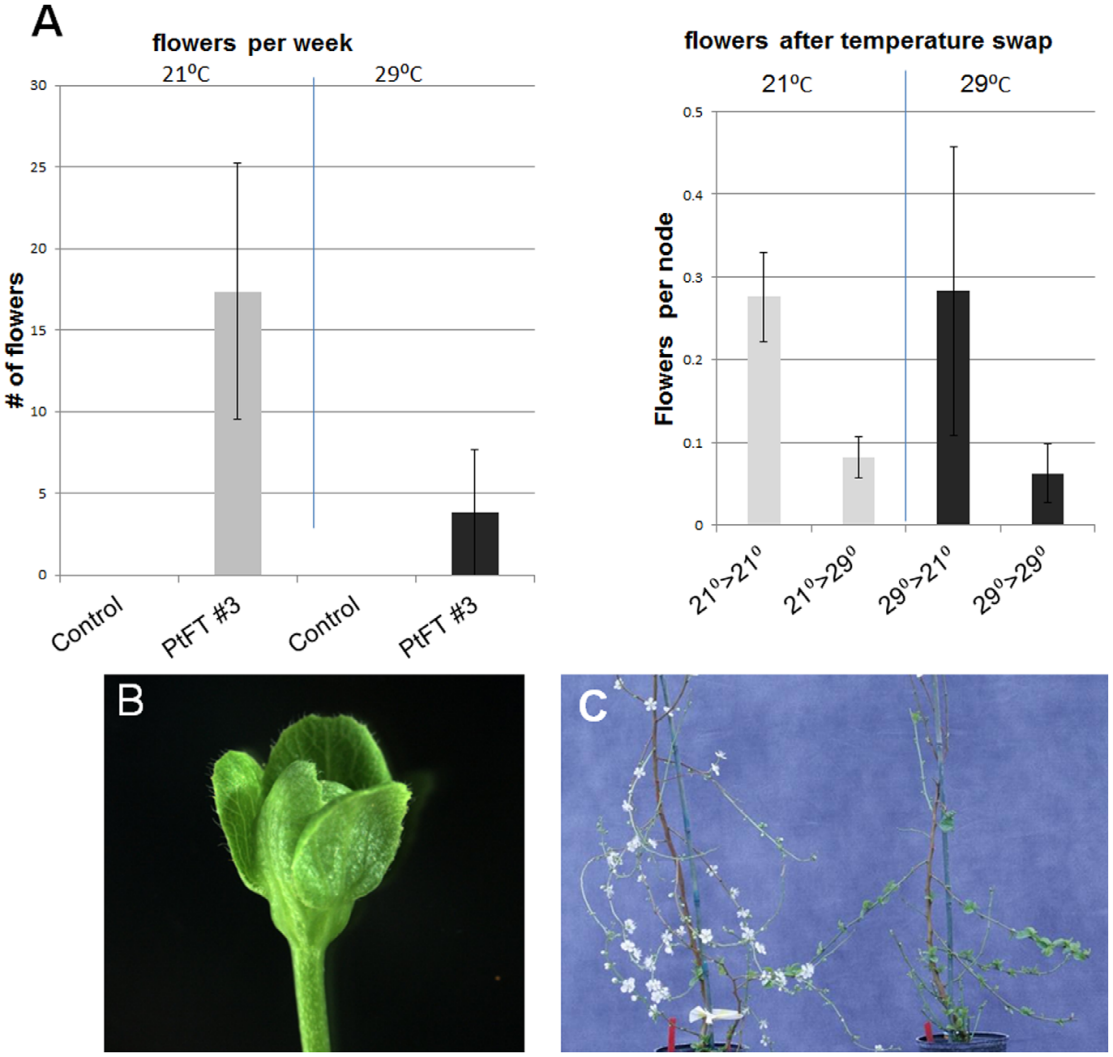
High temperature represses flowering. (A) Graphs showing differences in flowering of FT line #3 and control plums grown either under 21°C or 29°C. Y-axis represents number of flowers produced per week measured over a period of 3 weeks (left). After temperature swap, flowers were measured for a 3 week interval and shown as number per node (right). (B) Image of a typical abnormal leaf-like flower produced only at 29°C. (C) Image of flowering plants after temperature swap of FT line #3 either shifted or maintained at 21°C or 29°C. Error bars represent standard deviation.

Based on these field data, we investigated whether flowering in FT plums was predominantly controlled by temperature. Seasonal temperatures are known to play a key role in the growth and development of temperate trees. Normally, anthesis in plum occurs during early spring after which trees enter a vegetative growth phase in summer. It is during this summer growth period that new flower primordia are formed adjacent to lateral vegetative buds. To assess the influence of temperature on flowering, controlled temperature experiments were performed using environmental growth chambers. Twenty plants each of PtFT3 and non-transgenic controls were placed in environmental growth chambers at either 21°C or 29°C for eight weeks. New flowers, nodes, and buds that developed were counted at two week intervals for six weeks. Data showed that anthesis in FT plums occurred at both temperatures but was substantially more prolific at 21°C compared to 29°C (Fig. 6A), consistent with field observations.

To confirm the temperature effect, five plants from PtFT3 were interchanged between growth chambers after eight weeks. Anthesis increased in plants shifted from 29°C to 21°C and was repressed in plants shifted from 21°C to 29°C (Fig. 6C). In addition to changes in flowering, we also observed that higher temperature caused flower abnormalities in 10–30% of all flowers grown under 29°C. Abnormalities included shortened anthers and leafy petals and sepals (Fig. 6B). Overall, these experiments demonstrate that high temperature inhibits anthesis while lower temperatures promote anthesis. These results are consistent with natural plum growth dynamics during seasonal temperature cycles and suggest that over-expression of FT does not interfere with the normal temperature-mediated transition to floral bud formation.

FT is known to be a key integrator of flowering and photoperiod signaling pathways [1] [20]–[22]. The results presented here support these findings and further that the expression of FT affects apical dominance, stem growth and attachment, and inflorescence architecture. These broad impacts of FT suggest that it may play an even more fundamental role in plant growth and development than currently appreciated. Still, we cannot rule out that some of the observed changes are due to aberrations caused by use of the heterologous poplar *FT* gene. Irrespective of these possibilities, this work demonstrates that the developmental and ecophysiological properties of a tree species can be radically transformed in a useful fashion through the introduction of an *FT* transgene.

Over the next century world agriculture will face some of its greatest challenges. Population growth, climate change, and shrinking availability of agricultural land will demand dramatic increases in productivity. Crops engineered to adapt to alternative environments and/or new production schemes can play a vital role in that success. Currently, a number of fruit crops are amenable to this technology and other tree-based systems such as apple are current under development [11]–[12]. The ability of FT plums to continuously produce fruit regardless of day length or chilling time and still survive winter temperatures suggests they could be grown in both tropical and temperate climates. By continually flowering and setting fruit throughout the spring, losses from adverse weather events such as spring frosts could also be minimized. Their shrub-like growth habit potentially makes them suitable for novel production systems either in the field or protected facilities such as greenhouses or high tunnels. Thus, FT plums demonstrate that relatively simple biotechnological modifications can circumvent the natural ecophysiological barriers that restrict how, where, and when temperate tree fruits can be cultivated.

## Materials and Methods

### Plum Transformation

A total of 193 hypocotyl sections excised from 50 surface sterilized mature zygotic embryos of cv. Bluebyrd plum *(Prunus domestica* L) were transformed with *Agrobacterium tumefaciens* strain GV3101 [23] containing the *35S::PtFT1* [5]. The transformed hypocotyl sections were cultured *in vitro* and transgenic plants were regenerated and rooted as previously described [24]. The rooted plantlets were acclimated in the growth chamber (20°C; light intensity 70 µmol photons m2 s-1, 16 h light/8 h dark photoperiod; 70% relative humidity) for 2–3 weeks and planted in 6 or 9 inch pots containing Metro-Mix 510 (Sun Grow Horticulture Inc, Washington State, USA) and grown under sunlight in temperature controlled greenhouses (18–30°C). Data on tree architecture, flowering habit and time flowering were recorded. Since ‘BlueByrd’ plum is self-incompatible, FT plum flowers were manually pollinated with pollen collected from ‘Stanley’ plum.

### RNA Expression Analyses

RNA for expression analysis was extracted from a pooled sample (∼10 mg total) consisting of one leaf punch, using a standard paper punch, from three different young, fully expanded leaves from the same plant. The samples were frozen in liquid nitrogen and stored at –80°C until processed. All the leaf samples for one experiment were collected between 13∶00 and 14∶30 hours of the same day. RNA was extracted from the leaf material using the MagMAX-96 Total RNA Isolation Kit (Applied Biosystems, Foster City, CA) with some modifications to the protocol. Briefly, 100 µl of Lysis/Binding Solution was added to the sample along with 1/4 the standard amount of Lysing Matrix D (BIO 101 Systems, Thermo Scientific, Waltham, MA) and processed in a FastPrep (FP120, BIO 101, Thermo Scientific) bead beater for 27 sec at a 5 speed setting. The material was pelleted and the supernatant was placed in a clean microcentrifuge and processed as described in the manufactures protocol except that two washes were performed at each step. RNA was DNased using Turbo DNA-free kit (Applied Biosystems) following manufacturer’s directions. Three µl of the RNA was evaluated by gel electrophoresis.

Quantitative real-time PCR (qPCR) was performed on the RNAs utilizing a one-step protocol with RNase Inhibitor (Applied Biosystems), MuLV Reverse Transcriptase (Applied Biosystems) and SYBR Green PCR Master Mix (Applied Biosystems) following the manufacture’s protocol. The RNAs were run with and without reverse transcriptase, in order to verify the lack of significant DNA contamination. To determine the relative FT transcript levels, the RNAs were diluted (0.33 µl/reaction), and run in triplicate in 10 µl reactions on an ABI7900 (Applied Biosystems). Primer sequences are listed in Table 3. All RNAs were run with 28S primers at a 1000 fold dilution as a standard. A standard curve was run in triplicate with each primer set. The results of the triplicate reactions were averaged and normalized by the relative amount of 28S RNA. The relative amount of *PtFT* RNA was determined from the standard curve. To keep all the numbers on the same scale, the level of expression of the flowering line FT-126 was set at 100% and all the other lines were compared to that line. This experiment was repeated with recollected leaf samples with similar results (data not shown).

**Table 3:** Primer sequences used to amplify *PtFT* sequences in the transgenic plum leaves.

1PtFT1-5′	CAGAACTTCAACACCAGAGA
1PtFT1-3′	TCCTACCACCAGAGCCACT
228S-5′	GCAGCCAAGCCTTCATAGCG
228S-3	GTGCGAATCAACGGTTCCTC
34040-5′	CAAGGCAACTACAACTCAGGCAG
34040-3′	AGGCATCCCATACATAACACCAAG

1 Designed from *PtFT1* primers (bp415-517) as described in Bohlenius et al. (2006).

2 Previously published primers in Moon and Callahan (2004).

3 Designed from Contig 4040 in the *Prunus* Assembly V4 http://www.bioinfo.wsu.edu//cgi-bin/gdr/gdr_EST_contig_search.cgi?genus=Prunus based on Wu et al. (2006).

### Determination of Relative PtFT Gene Copy Number

To determine copy number of the transgene *PtFT*, DNA was extracted using a modified CTAB protocol (Doyle and Doyle, 1988) and subjected to real time PCR utilizing the SYBR Green PCR Master Mix (Applied Biosystems). Primers *PtFT* -5′ and 3′ are specific to the inserted *FT* gene and primers 4040 -5′ and 3′were used as the baseline for a single copy gene. A standard curve was run for each set of primers. The relative value for each transgenic line was determined from the curve and the ratio of *PtFT* to the standard single copy gene, 4040, most closely related to an unknown protein in *Arabidopsis*, AT1G13380 was determined [25]. The lowest ratio was set to one copy and the remaining ratios were divided by that number to determine the relative copy number.

### Cold-induced Dormancy

One year old clonally propagated FT plum plants from transgenic lines #3 and #34 along with control plums (non-FT progeny of the same parent ‘Bluebyrd’) were stripped of all leaves and flowers and placed into a cold room (5°C) without light. Three or four plants from each transgenic line and controls were removed at one or two week intervals and placed in the greenhouse to promote growth. Bud break was scored once per week for four weeks and the rate of bud break was calculated as the number of buds that grew out relative to the total number of buds on each plant.

Survival of field buds was measured from bud sticks collected in late January. 100 vegetative buds from 3 plants collected from each clone were sectioned with a scalpel to evaluate survival rates. Those that had turned brown and desiccated were considered non-viable while those that were green and succulent were scored as having survived.

### Temperature and Day Length Experiments

Clonally propagated FT plums and control plums (non-FT seedlings from the same parent ‘Bluebyrd’) were divided into two groups to be grown in matching growth chambers with a mix of incandescent and fluorescent lighting resulting in ∼900 µmol photons m^2^ s-1 of light measured at the average plum canopy. In the temperature experiment both chambers were set for 16 hr light with 8 hr of dark at 50% humidity. One chamber was set at 21°C and the other 29°C. Ten FT plants from line #3, line # 34 and line #157 were placed in each chamber. Plants were measured at the start of the experiment and every two weeks thereafter for a total of 8 weeks of growth. Five plants of line #3 were exchanged–moved from 21°C to 29°C and five plants were moved from 29°C to 21°C. Bud formation and break were scored on those moved plants and the five plants that had not been moved. For day length experiments, plants were placed in two separate growth chambers set for a 16 hr or 8 hr photoperiod, respectively. Temperature settings in each chamber were optimized to maintain 21°C and 50% humidity. Plants were stripped of all leaves, terminal buds were removed and laterals were pruned to 10 cm in length. Laterals producing terminal buds were counted after 3 weeks, 5 weeks and 7 weeks. At that time the plants were shifted to the reciprocal chamber and measurements were taken after two and four weeks.
